# Report on Therapeutics

**Published:** 1876-08

**Authors:** F. Peabody


### REPORT ON THERAPEUTICS.

BY F. PEABODY, D. D. S.

*Read before the Kentucky State Dental Association.*

Your committee had concluded, up to June 4th, 1876, the day before the opening of this association, to make no report on dental therapeutics; but a slight circumstance has induced them to hastily throw together a few ideas, more for the purpose of eliciting discussion than for anything else. If the members of this association expect to learn anything new, I fear such expectations will not be realized. I propose to touch but lightly on some of the more prominent remedies, and perhaps bring to mind some which may have been forgotten or laid aside.

The field of general therapeutics is a broad one, too broad to have more than a passing glance given to that portion relating more directly to dentistry in a paper as hastily written as this must necessarily be.

Broad though the field is, yet that portion applied directly to our calling is exceedingly limited. It is much to be regretted that more attention is not paid to this branch in the curriculum of our dental colleges, and that our text books are not in this respect better adapted to our wants. But the list of acceptable remedies is, year by year, being extended; and it is to be hoped that we shall soon have at our command as extensive a materia medica in proportion to our acquirements, as has the physician in general practice for his.

As we acquire a better knowledge of the pathology of our profession so must our understanding be enlarged as to the means of overcoming pathological conditions, and our knowledge of the agents necessary to make physiological out of pathological organs must necessarily be increased. It is gratifying to know that light is constantly being thrown on the darkness that has so long surrounded us, that our progrsss is less groping and stumbling, that we can now with more certainty pronounce what results will follow our treatment of the various classes of disease, than we have heretofore been enabled to do. It is not so many years since pathological conditions of the mouth and teeth, which we now successfully combat, were considered beyond the pale of our knowledge and treatment; and as year by year we are advancing in the right direction, and adding to our store of information, we may with confidence look forward to the time when we may be considered as having through our researches and the results attending them, earned the right to stand side by side with the foremost branches of medical science. Our profession as a profession is yet in its infancy; but, from the rapid strides that have been made during the last decade of years, we may reasonably hope to attain to full growth in a much shorter time than has been required for most other professions. As a profession our knowledge is to much limited to topical agents and topical applications. We have not become sufficiently familiar with the systemic causes that often underlie the diseases we have to treat, and as a consequence, our knowledge is less pronounced as to systemic remedies. This is to be regretted; but time and patient investigation, close observation, and a record of our failures as well as our successes will ultimately lead into paths of wisdom. In our associations we do not discuss as we should, the subject of therapeutics; we hear more of general principles that are the ground work of our specialty, or of operations and their methods, than of our dental materia medica. Those who have a greater insight and a larger experience on this subject, do not shed on it sufficient light to illumine our pathway; in consequence our efforts for the present, must to some extent be empirical. We have in many cases, so little data to rely upon that we must depend largely on experiments, and the results of experience and observation, or turn our patients over to the general practitioner, where they often fare worse than had we given them the benefit of such knowledge as we possess. A notable instance of which came under my observation in a patient who had been treated by a physician for some time for deafness, without benefit, the whole cause of which was found to be in one of the wisdom teeth, which on being removed gave immediate relief from pain, and at once restored the hearing. This is but one of the many instances that might be cited, where physicians work as much in the dark as we do; particularly where the disease belongs more properly to our specialty.

By therapeutics is meant the appreciation, as well as the discovery of medicines. Many valuable agents have been pronounced worthless by those who have not found success in their use, simply because the manner of their application was not well understood. As an instance, note the various opinions as to the results attending the use of oxychloride of zinc. In the hands of some, it is pronounced as perfectly successful, while others meet with little else than failures in its use. Now it is hardly reasonable to suppose that the success of one was the result of his having only a class of patients whose temperaments were such as would tolerate this agent in any form, no matter how used, and that the patients of the other had temperaments of an entirely opposite character. It is in the manipulation and application and the knowledge of when and how to use it that we find such results as we are seeking

As a non-conductor under a metallic filling, perhaps nothing is better than oxychloride of zinc, and yet this does not answer our purpose so much better than other things, simply because it is a non-conductor, but because it can be so readily introduced and because it adapts itself so thoroughly to all parts of the cavity without pressure. It has been ascertained, and correctly, that a healthy, freshly exposed pulp, will not in all mouths tolerate this substance, on account of the caustic character of the chloride, and in order to still use it as a non-conductor without risk of destroying the pulp, something of a less irritating character must intervene between this highly sensitive and delicate organ and the inflammatory action of the liquid of the compound, thus making it useful by a knowledge of its properties and the manner of controling them.

The past year, as far as your committee are aware, has given us nothing new in dental medicines. Salicylic acid in place of carbolic acid has been attracting the attention of the profession. It has hardly been in use long enough to pronounce positively in regard to its value. It is a powerful disinfectant, more so than carbolic acid, and has the advantage of being odorless. It is not readily dissolved in pure water, though easily dissolved in ether or alcohol. It is often more difficult to introduce into the canal of a tooth or a cavity in its dry state than when in solution. It is believed that better results can be obtained with it than from carbolic acid, but in some instances the result has not justified this. With some it is a favorite remedy, while others have not met with the success in its use they had anticipated, perhaps its properties as yet are not sufficiently understood for us to make the best use of it, it may be found to act better in connection with some other agent than by itself.

The tincture of callendula is one of the most valuable articles in our list of remedies. It is not as well known to the profession as some other agents, and, strange to say, is not generally known to the allopathic pharmacy, though it can be obtained of any homœopathist. It is made from the flowers of marygold, and was formerly used internally as a medicine, but fell generally into disuse. The allopathic school seems to have overlooked it as a topical agent. Probably callendula has no superior as an application to abraded surfaces or wounds, and is one of the best mouth washes known particularly after the extraction of teeth. When used for this purpose it should be diluted with four or five parts of water; for other wounds it should be used in its full strength.

"The dental pain obtunder" has been in use for something over a year; the opinions of its value seem to be numerous. In some cases it acts well, while in others it produces no effect. It does not, on the whole, seem to be entitled to any particular merit over other recognized applications for sensitive dentine. Nothing pronounced has been made known to the profession that will act well in all cases to mitigate this sensitiveness of the tooth bone. Acetate of morphine with carbolic acid acts well in some cases. Chromic acid, chloride of zinc, tincture of aconite each gives good results, and sometimes oil of peppermint will answer where other articles fail. The use of arsenous acid for this purpose, though never failing, can not meet the approbation of your committee. Inducing the patient to take a deep inspiration and retain the breath, and while the breath is being retained, if the operator will with a sharp excavator cut rapidly for fifteen or twenty seconds, then give the patient a moment's rest and repeat, he will find that he can excavate sensitive teeth with more ease to the patient and more rapidly than he can while the tooth is under the influence of most of the obtundants. This is more positively marked if the cavity be kept perfectly dry. Where there is exalted sensibility, and no haste is necessary, filling the tooth with oxychloride of zinc and allowing it to remain six weeks or two months will, very often, enable the operator to remove decay, and shape cavities to his satisfaction that before would hardly tolerate the touch of an instrument. Carbolic acid alone sometimes produces the best of results in allaying the sensitiveness of dentine. In acute inflamation of the nerve, resulting from the pressue of a filling or other causes that produce congestion, an old and but little used practice of drilling through the alveolus into the pulp canal until the root vessels are punctured and relieved of their overcharged blood will often give immediate relief. This is a delicate operation, and the operator must understand well the conditions attending the case, and his instrument must be of the right shape and carried with a steady hand, or the death of the pulp will be the result. It is also an exceedingly painful operation, and few patients are willing to submit to it the second time. Local applications of equal parts of compound tincture of iodine and tincture of aconite, will often give relief, both in congestion of the pulp, and in periosteal inflammation. In periodontitis, homœopathic doses of antimonium crudum (one grain every two hours) often subdues the pain, but in some cases the application of a leech, to relieve the engorged condition of the parts, will be found necessary.

Veratria in glycerine will generally give immediate ease to acute pains in the teeth whether from exposed pulp or inflammation of the membrane. It acts as a counter irritant and should be rubbed on the gums over the apex of the offending tooth; but a very small portion should be used a quantity not larger than the head of a pin Tincture of aconite alternated with chloroform will often help, but where the aconite or veratria are used, great care should be exercised that none of the saliva be swallowed, as they are poisonous, and the veratria is apt to produce excessive nausea. In treating alveolar abscess, alcohol alone will often be all that is necessary. A saturated solution of matallic iodine in wood creosote is almost a certain cure in acute cases provided the preparation thoroughly reaches all parts of the sac. Where there is no external opening, one should be made with a drill at the apex of the root, as the air in the canal will act as an elastic cushion, keeping the medicine from reaching the end of the root and passing through it, and unless it does so we can never be sure that the caustic has reached the part designed.

In chronic abscesses iodoform is highly recommended, it is rubbed up in glycerine and thoroughly pumped through the roots by a few fibres of cotton wrapped around a broach. Where this has failed success has attended the use of lead wire for a filling for the roots, as explained at the last meeting of this association. All *debris* should be thoroughly removed from the canal; carbolic acid or better still, pine wood creosote should then be used, pumping it through the fistulous opening after inflammation has been reduced as much as possible, the roots should be thoroughly filled with lead wire, the first piece rolled to a fine point the size of the foramen, but not so small as to pass through it. The most gratifying results have attended this practice, and some of the most obstinate cases have succumbed to this treatment. It is not claimed that it will cure *all* cases, but fewer failures have been met with, when thus treated, than from any other method, as far as has been learned.

In all cases the surrounding conditions should be carefully observed, and taken into consideration, and our practice should be governed by the temperament and idiosyncrasy of the patient.

Tannic acid following the use of arsenous acid in devitalizing a pulp, if allowed to remain a few weeks, will so thoroughly convert the pulp into a tannate that it can be removed with absolutely no pain. This is a great disideratum, as the pulp comes from the tooth perfectly desiccated, and leaves no particles to give future trouble from decomposition and generation of gas. There are many nice points in the practice of our profession, which would be of great advantage to all, were they thoroughly understood; but enough has been said in this paper to call forth from each member of this association some affirmation or refutation of what it contains; and if it will do that, the object it aims at will have been accomplished.

